# EUS-FNA for the Diagnosis of Retroperitoneal Primitive Neuroectodermal Tumor

**DOI:** 10.1155/2011/198029

**Published:** 2011-04-06

**Authors:** Aijaz A. Sofi, Ashish D. Thekdi, Ali Nawras

**Affiliations:** ^1^Division of Gastroenterology, Department of Internal Medicine, University of Toledo Medical Center, 3000 Arlington Avenue, Toledo, OH 43614, USA; ^2^Division of Gastroenterology, Department of Internal Medicine, Henry Ford Health System, Henry Ford Hospital, Detroit, MI 48202, USA

## Abstract

Primitive neuroectodermal tumor (PNET) is a rare “small round blue cell tumor” that is diagnosed by open biopsy or percutaneous biopsy of the lesion under radiologic guidance. In this case report, we present a novel approach to the diagnosis of a retroperitoneal PNET by endoscopic ultrasound- (EUS-) guided fine needle aspiration (FNA). A 35-year-old man presented with the history of left-sided flank pain and swelling of 3-weeks duration. Computerized tomography (CT) scan of his abdomen revealed a 12.8 × 13 × 12.5 cm cystic and solid mass arising from the retroperitoneum and displacing the third and fourth portions of the duodenum. He underwent EUS which revealed a well-circumscribed heterogeneous mass abutting the inferior portion of the stomach. EUS-FNA of the mass revealed malignant cells consistent with primitive neuroectodermal tumor (PNET)/Ewing's sarcoma. EUS-guided FNA is an appropriate technique for diagnosing retroperitoneal PNET/Ewing's sarcoma.

## 1. Introduction

Endoscopic ultrasound- (EUS-) guided fine needle aspiration (FNA) is a widely used diagnostic method for obtaining tissue samples from lesions within the gastrointestinal (GI) tract and in select extra-GI conditions such as for sampling of hilar tumors, mediastinal lymph nodes, gallbladder lesions, pancreatic lesions, and kidney/adrenal masses. Primitive neuroectodermal tumor (PNET) is a rare “small round blue cell tumor” that belongs to the family of Ewing's sarcoma [1]. Diagnosis of this condition is routinely based on obtaining pathological samples by open biopsy or core biopsy and, recently, the FNA technique. We report a case wherein a retroperitoneal PNET was diagnosed after biopsy using EUS-guided FNA.

## 2. Case Presentation

**Figure 1:**
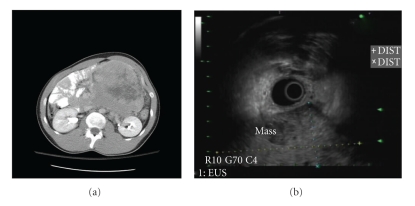
(a) CT film showing large mass displacing small bowel to the right. The mass measured 12.8 × 13 × 12.5 cm arising from the retroperitoneum and was displacing the 3rd and 4th portions of the duodenum to the right. (b) EUS image of large hypoechoic mass adjacent to the gastric wall, measuring 9.6 × 7.4 cm. The distal aspect of the mass was beyond the reach of the ultrasound probe.

**Figure 2:**
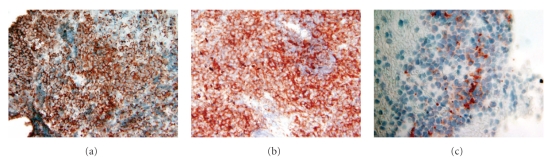
Immunostains performed on cell block material displaying immunoreactivity of tumor cells with CD 99 (a), CD 117 (b), and synaptophysin (c).

A 35-year-old man of middle-eastern origin presented with left flank pain for 3 weeks. He had no significant past medical or surgical history. Review of systems was significant for change in bowel habits (constipation), weight loss (12 lbs in one month), and early satiety. Physical examination revealed large palpable, nontender left abdominal mass. Laboratories showed normocytic anemia with hemoglobin of 12.2 g/dL and mildly elevated level of alpha-fetoprotein (14.6 ng/ml). Computerized tomography (CT)-scan of the abdomen revealed a 12.8 × 13 × 12.5 cm cystic and solid mass displacing the third and fourth portions of the duodenum to the right (Figure 1(a)). EUS was performed which revealed a large hypoechoic mass, with internal anechoic areas and well-demarcated borders adjacent to the gastric wall (Figure 1(b)). Maximum diameter of the mass on EUS was 9.6 × 7.4 cm, but the outer border of the mass was beyond the limit of ultrasound penetration depth. EUS-FNA was performed using a 22-gauge needle, and a total of 5 passes were performed. Bedside cytopathology confirmed adequacy of specimen. Microscopic examination of cytologic material revealed a cellular specimen composed of numerous round blue cells arranged singly and as loosely cohesive clusters. Individual tumor cells displayed enlarged hyperchromatic nuclei and a relatively scant cytoplasm. Immunostaining of cell block material revealed immunoreactivity for CD99 (Figure 2(a)), c-kit (Figure 2(b)), and synaptophysin (Figure 2(c)). Immunoreactivities for leucocyte common antigen, epithelial membrane antigen, pancytokeratin, and chromogranin were all negative in tumor cells. The combined cytomorphology and immunophenotype were consistent with a peripheral neuroectodermal tumor/Ewing's sarcoma. Further imaging with positron emission tomography (PET) revealed no evidence of metastatic disease. The patient was started on neoadjuvant chemotherapy with a 5-drug regimen: Vincristine, Adriamycin, Cytoxan, Ifosfamide, and Etoposide. Repeat CT scan six weeks after chemotherapy revealed shrinking of the lesion to a size of 8.4 × 7.3 × 9.0 cm with central necrosis. He underwent exploratory laparotomy with complete excision of retroperitoneal tumor. The diagnosis of PNET/Ewing's sarcoma was subsequently confirmed by histopathology of the excised tumor. 

## 3. Discussion

Ewing's tumor arises from long bones and soft tissue. When Ewing's tumor arises from soft tissues, it is called “extraskeletal Ewing's tumor” (EES). PNET, similar to Ewing's tumor is a round cell tumor originating from neuroectodermal crest. Histologically, EES and PNET are closely related tumors and have been grouped together as Ewing family of tumors [1]. Patients with abdominal/retroperitoneal PNET present with nonspecific symptoms like pain and a palpable mass on examination. Diagnosis is routinely based on imaging studies; however, biopsy is essential for definitive diagnosis. The most commonly utilized biopsy techniques are either open biopsy or an imaging-guided core biopsy. FNA has not classically been used due to smaller tissue sample and lack of tissue architecture. However, several studies have established the usefulness of FNA in providing accurate diagnosis through the new technology of immunocytochemistry, DNA flow cytometry and molecular genetic studies [2–4]. 

As reported in this case, large tumors frequently need to undergo neoadjuvant chemotherapy for tumor shrinkage and thereby better surgical resection. Therefore, obtaining precise diagnosis is fundamental for initiating proper chemotherapeutic regime. Furthermore, the use of minimally invasive technique in the diagnosis is preferred as it minimizes the procedure-related risk and danger of malignant cell dissemination during percutaneous biopsy. The reported incidence of needle tract seeding ranges between 0.003% and 0.009% [5]. Tumor seeding of EUS-FNA tract has been sparsely reported in the literature [6, 7], and substantial evidence for this complication is still lacking. Notably, the risk of peritoneal spread of tumor cells is much lower with EUS-FNA compared to percutaneous FNA [8]. 

Retroperitoneal PNET is uncommon, and evidence for preferred diagnostic modalities for these lesions is not established. However, based on the available evidence and clinical reasoning, EUS-guided FNA appears a preferable approach to diagnose retroperitoneal tumors including PNET. The location of the tumor may further be a factor in determining the preferred diagnostic approach. If the tumor is in the proximity of the GI tract, EUS-guided FNA may be a preferred approach. 

EUS-guided FNA is an established method of obtaining tissue samples for diagnosis of lesions within the GI tract as well as outside. [9]. To the best of our knowledge, this would be the first detailed documentation providing evidence for diagnosing retroperitoneal PNET using EUS-guided FNA [10]. Our case demonstrates that EUS-guided FNA is an emerging modality with potential to diagnose several lesions outside of the GI tract.
